# Suivi thérapeutique pharmacologique de trois médicaments antiépileptiques: retour sur vingt années d’expérience

**DOI:** 10.11604/pamj.2016.25.10.9664

**Published:** 2016-09-19

**Authors:** Samira Serragui, Fatima Zalagh, Driss Soussi Tanani, Lahcen Ouammi, Latifa Ait Moussa, Narjis Badrane, Rachida Soulaymani Bencheikh

**Affiliations:** 1 Laboratoire de Pharmacologie et de Toxicologie, Faculté de Médecine et de Pharmacie, Université Mohammed V, Rabat, Maroc; 2 Centre Anti Poison et de Pharmacovigilance du Maroc; 3 Laboratoire de Pharmacologie Faculté de Médecine et de Pharmacie, Université Abdelmalek Essaadi, Tanger, Maroc

**Keywords:** Antiépileptiques, suivi thérapeutique pharmacologique, phénobarbital, Antiepileptics, Therapeutic drug monitoring

## Abstract

**Introduction:**

Le suivi thérapeutique pharmacologique (STP) des médicaments antiépileptiques (MAE) est un outil très utilisé dans la gestion de l'épilepsie. Au Maroc, ce dosage est réalisé au Centre Anti Poison et de Pharmacovigilance du Maroc (CAPM) depuis Avril 1995.

**Méthodes:**

Il s'agit d'une étude rétrospective s'étalant sur 20 ans. Elle concerne le STP du Phénobarbital (PB), de la Carbamazépine (CBZ) et de l'Acide Valproique (AVP).

**Résultats:**

Le STP des 3 MAE représentaient 58,85% de l'ensemble des demandes de STP reçue par le CAPM. Le dosage du PB était classé en première position suivi par celui de la CBZ et enfin par l'AVP. La faible demande de STP au Maroc pouvait être expliquée par le faible nombre de neurologues auquel s'ajoutaient des facteurs sociaux. Grâce à son prix très accessible par les patients, le PB est le MAE le plus prescrit dans notre pays expliquant ainsi la demande élevée de son dosage. Quant aux motifs de STP des 3 MAE, ils étaient essentiellement liés à l'âge, à l'apparition d'effets indésirables, à l'association de MAE ou dans le cas de vérification de l'observance des malades.

**Conclusion:**

Des efforts sont à fournir pour promouvoir l'intérêt du STP des MAE dans la prise en charge de l'épilepsie au Maroc.

## Abstract (en)

**Introduction:**

The therapeutic drug monitoring (TDM) of antiepileptic drugs is a tool widely used in the management of epilepsy. In Morocco, this monitoring is carried out by the Centre Anti Poison et Pharmacovigilance (CAPM) since April 1995.

**Methods:**

This is a retrospective study spanning 20 years. It concerns the therapeutic drug monitoring of Phenobarbital (PB) of carbamazepine (CBZ) and valproic acid (VPA).

**Results:**

Therapeutic drug monitoring of the 3 antiepileptic drugs represent 58.85% of all applications received by the CAPM. The dosage of PB was ranked first followed by that of CBZ and finally by the VPA. Weak demand for therapeutic drug monitoring in Morocco could be explained by the low number of neurologists in addition to social factors. With its affordable price by patients, PB is the most prescribed antiepileptic drug in our country, which explains the high demand for its dosage. As for the therapeutic drug monitoring of the antiepileptic drug, they were mainly related to age, the occurrence of adverse effects, the association antiepileptic drugs or in the case of verification of patient compliance.

**Conclusion:**

Efforts are required for promoting the interests of therapeutic drug monitoring of antiepileptic drug in the management of epilepsy in Morocco.

## Introduction

L'épilepsie est une maladie très fréquente [1–5]. Au Maroc, elle est en deuxième position après la migraine. Sa prévalence est estimée à 1,1 % [6]. Depuis les années quatre-vingt-dix, avec l'augmentation du nombre de neurologues marocains, la prise en charge des malades épileptiques a connu de grandes avancées. Ainsi, parmi les outils utilisés pour mieux gérer les effets indésirables liés aux médicaments antiépileptiques (MAE) et guider le médecin dans son évaluation de la non-compliance et des variations interindividuelles, il y a le suivi thérapeutique pharmacologique (STP) des MAE [7–13]. A Rabat, depuis Avril 1995, ce dosage est assuré par le laboratoire du Centre Antipoison et de Pharmacovigilance du Maroc (CAPM) qui dispose d'une unité spécialisée en STP. L'objectif de notre étude est de faire une évaluation des demandes de STP de 3 MAE majeurs à savoir le phénobarbital (PB), la carbamazépine (CBZ) et l'acide valproique (AVP) qui ont été traitées par le CAPM sur une période de 20 ans.

## Méthodes

Il s'agit d'une étude rétrospective concernant les demandes de STP du PB, de la CBZ et de l'AVP réalisées au niveau du laboratoire du CAPM entre Avril 1995 et Avril 2015. Etant donné que le prélèvement des échantillons sanguins est la phase cruciale du STP sur laquelle repose l'interprétation correcte des concentrations sanguines des médicaments, le CAPM a développé des procédures de prélèvement sanguin respectant toutes les conditions indispensable au STP des 3 MAE. Le dosage a été effectué par la Chromatographie liquide haute performance avec un détecteur UV/Visible à longueur d'onde variable avec une extraction liquide-liquide [14–21]. L'évaluation de STP de ces 3 molécules a été basée sur la détermination de son pourcentage par rapport aux autres médicaments dosés et sur leur répartition pour chacun des 3 MAE.

## Résultats

**Figure 1:**
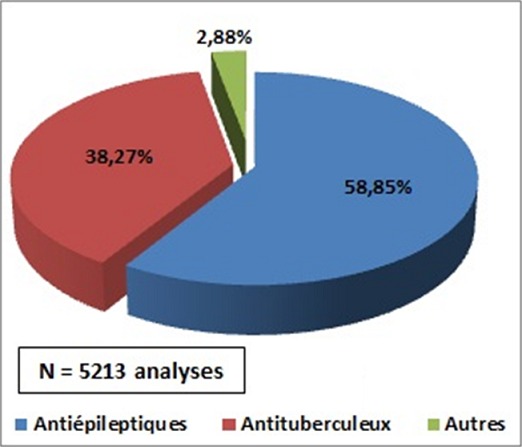
Place du STP des antiépileptiques par rapport au STP des autres molécules dosés au CAPM

**Figure 2:**
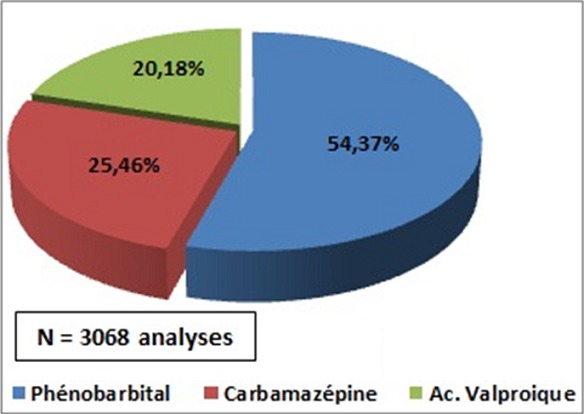
Répartition des 3 antiépileptiques en fonction de pourcentage de leur dosage

En 20 ans, l'unité de STP du laboratoire du CAPM a assuré 5213 dosages dont 58,85% correspondaient aux PB, CBZ et AVP (Figure 1). Les demandes concernaient des patients de tous les âges. Elles provenaient essentiellement du Centre Hospitalo-universitaire de Rabat. La répartition des demandes montraient que le PB était en première position avec 54,37% dosage suivi par la CBZ avec 25,46% et l'AVP avec 20,18% (Figure 2). La répartition de dosage durant ces 20 années montrait que les demandes de dosage du PB variaient entre 1 et 178, celles du CBZ entre 3 et 142 et de l'AVP entre 0 et 59 (Tableau 1). Les motifs des demandes étaient en cas d'apparition d'effets indésirables, d'échec thérapeutique, d'association de ces MAE ou de vérification de la compliance des patients.

**Tableau 1:** Répartition du STP des 3 MAE en fonction des molécules

Nombre de dosages			
	PB	CBZ	AVP
N	1668	781	619
Médiane	69	23	28
Moyenne	79,43	37,19	29,48
Ecart type	62,70	38,06	19,55
Min	1	3	0
Max	178	142	59

## Discussion

En 20 ans, le nombre de demandes de dosage reçu par le CAPM reste très faible par rapport au nombre de patients épileptiques marocains qui est estimé entre 160 000 et 300 000 [6]. Cette faible demande de STP ne peut être expliquée que par un effectif réduit de médecins traitants. En effet, malgré les efforts fournis ces dernières années dans la formation des neurologues, leur nombre reste toujours insuffisant puisqu'il n'est que de 120 [22]. A ce problème s'ajoute celui de la couverture sociale qui n'est pas généralisée à tous les patients épileptiques ce qui rend très difficile leur prise en charge [23]. A ces facteurs économiques s'ajoutaient des facteurs sociaux. Ainsi, à cause de l'analphabétisme, le manque de culture médicale et le poids des traditions, la plupart des patients épileptiques faisaient appels aux tradipraticiens et ne concevaient pas l'intérêt de se faire prélever pour adapter leur traitement ce qui est à l'origine de la perte de plusieurs demandes de STP [24].

Quant à la prescription des MAE au Maroc, le PB se trouve en tête. Bien que la plupart des médicaments antiépileptiques soient disponibles, le déterminant majeur qui gère leur prescription reste le coût du traitement [25]. Pour son prix très accessible pour les patients marocains, le PB reste l'antiépileptique le plus prescrit dans notre pays expliquant ainsi la plus forte demande de son dosage.

Un des facteurs qui peut être à l'origine des modifications des concentrations des MAE justifiant ainsi leur demande de STP, il y a l'âge du patient. Ce facteur peut être à l'origine des modifications du métabolisme et de l'élimination [26–29]. Le STP permettra alors aux cliniciens d'avoir une idée sur la marge de manœuvre leur permettant de faire varier les concentrations tout en évitant la survenue des effets secondaires. En effet, une partie des patients pour qui le dosage a été demandé étaient des enfants et des sujets âgés. Nos résultats sont en accords avec ceux de Battino et ses collaborateurs qui ont réalisé une étude chez un groupe de patients épileptiques âgés de plus de 65 ans [30]. Ces malades étaient sous carbamazépine. Les résultats du STP de ce médicament ont montré une diminution de la clairance de ce médicament de 23% par rapport au groupe témoin dont l'âge est compris entre 20 et 50 ans justifiant ainsi l'intérêt du STP des MAE chez les sujets âgés. Une autre étude réalisée par Battino et ses collaborateurs chez les enfants épileptiques a bien montré l'importance d'adopté le STP des MAE chez cette catégorie de patients [31].

Les motifs des demandes du dosage du PB, de la CBZ et de l'AVP, reçu par le CAPM, correspondaient aux différents niveaux de recommandations du STP. La survenue des effets indésirables était le motif le plus fréquent. Le dosage de ces 3 antiépileptiques a permis de savoir si le médicament en question est imputé. De cette façon, le STP a aidé le clinicien au diagnostic de toxicité surtout chez les patients dont le statut était difficile à évaluer cliniquement comme les jeunes enfants et les sujets souffrant de troubles mentaux [32]. Une fois la dose est ajustée, le clinicien pourra connaître la concentration plasmatique efficace propre au patient et constituer une référence individuelle qui pourra l'aider à mieux gérer les prises de décisions face à une modification de l´état clinique se produisant au fil du temps [33–37].

Les interactions médicamenteuses qui peuvent avoir lieu en cas bi ou trithérapie avec ces MAE, justifient les demandes de STP de ces molécules [38–41]. En effet, Fukuoka et al. [42] ont réalisé une étude chez 119 patients épileptiques sous carbamazépine seule, 91 patients en bithérapie soit avec le phénobarbital ou avec la phénytoine et 64 malades en polythérapie avec les 3 antiépileptiques. Ces auteurs ont montré que le phénobarbital fait diminuer la concentration de la carbamazépine d'un facteur de 0,77.

La vérification de l'observance des patients était aussi un des motifs du STP des 3 molécules. L'étude Specht et al. [43] l'a aussi montré puisque 44% des 52 patients inclus dans leur travail avaient des concentrations plasmatiques inférieures à plus de 50% de la concentration individuelle de référence. La non compliance de ses malades était la cause de cette diminution de concentrations. D'autres travaux ont apporté que les adolescents et les jeunes adultes constituent un sous groupe à risque plus élevé pour la non observance [44, 45].

## Conclusion

Au Maroc, avec la stratégie adoptée dans le domaine de la formation, le nombre de médecins neurologues est en nette progression et la demande de STP des MAE suivra puisque l'intérêt que peut porter le STP dans le management de l'épilepsie est intégré dans les modules de leur formation. Cependant, il reste des efforts à fournir auprès de la population pour éviter les pertes des demandes de STP liées aux croyances et à l'utilisation de médecines alternatives.

### Etat des connaissances actuelles sur le sujet

- Le suivi thérapeutique pharmacologique des antiépileptiques est un moyen de gestion des effets indésirables liés à ces médicaments;
- L'observance des patients au traitement antiépileptique peut être contrôlée par le suivi thérapeutique pharmacologique;
- Les changements des concentrations plasmatiques des antiépileptiques qui sont dus à l'âge ou à d'autres facteurs physiologiques ou pathologiques peuvent être gérer par le suivi thérapeutique pharmacologique.

### Contribution de notre étude à la connaissance

- Expérience du Maroc dans le suivi thérapeutique pharmacologique des antiépileptiques;
- Place du suivi thérapeutique pharmacologique des antiépileptiques dans la pratique quotidienne des cliniciens marocains.
